# A ketone monoester drink reduces postprandial blood glucose concentrations in adults with type 2 diabetes: a randomised controlled trial

**DOI:** 10.1007/s00125-024-06122-7

**Published:** 2024-03-14

**Authors:** Alistair J. Monteyne, Kaja Falkenhain, Gráinne Whelehan, Helena Neudorf, Doaa R. Abdelrahman, Andrew J. Murton, Benjamin T. Wall, Francis B. Stephens, Jonathan P. Little

**Affiliations:** 1 Nutritional Physiology Research Group, Department of Public Health and Sport Sciences, University of Exeter, Exeter, UK; 2 School of Health and Exercise Sciences, University of British Columbia Okanagan, Kelowna, BC Canada; 3 Department of Surgery, University of Texas Medical Branch, Galveston, TX USA; 4 Sealy Center on Aging, University of Texas Medical Branch, Galveston, TX USA

**Keywords:** Carbohydrate metabolism, Clinical science, Human, Insulin resistance, Insulin sensitivity, Metabolic physiology in vivo, Nutrition and diet, Oral pharmacological agents

## Abstract

**Aims/hypothesis:**

The aim of the present study was to conduct a randomised, placebo-controlled, double-blind, crossover trial to determine whether pre-meal ketone monoester ingestion reduces postprandial glucose concentrations in individuals with type 2 diabetes.

**Methods:**

In this double-blind, placebo-controlled, crossover design study, ten participants with type 2 diabetes (age 59±1.7 years, 50% female, BMI 32±1 kg/m^2^, HbA_1c_ 54±2 mmol/mol [7.1±0.2%]) were randomised using computer-generated random numbers. The study took place at the Nutritional Physiology Research Unit, University of Exeter, Exeter, UK. Using a dual-glucose tracer approach, we assessed glucose kinetics after the ingestion of a 0.5 g/kg body mass ketone monoester (KME) or a taste-matched non-caloric placebo before a mixed-meal tolerance test. The primary outcome measure was endogenous glucose production. Secondary outcome measures were total glucose appearance rate and exogenous glucose appearance rate, glucose disappearance rate, blood glucose, serum insulin, β-OHB and NEFA levels, and energy expenditure.

**Results:**

Data for all ten participants were analysed. KME ingestion increased mean ± SEM plasma beta-hydroxybutyrate from 0.3±0.03 mmol/l to a peak of 4.3±1.2 mmol/l while reducing 2 h postprandial glucose concentrations by ~18% and 4 h postprandial glucose concentrations by ~12%, predominately as a result of a 28% decrease in the 2 h rate of glucose appearance following meal ingestion (all *p*<0.05). The reduction in blood glucose concentrations was associated with suppressed plasma NEFA concentrations after KME ingestion, with no difference in plasma insulin concentrations between the control and KME conditions. Postprandial endogenous glucose production was unaffected by KME ingestion (mean ± SEM 0.76±0.15 and 0.88±0.10 mg kg^–1^ min^–1^ for the control and KME, respectively). No adverse effects of KME ingestion were observed.

**Conclusions/interpretation:**

KME ingestion appears to delay glucose absorption in adults with type 2 diabetes, thereby reducing postprandial glucose concentrations. Future work to explore the therapeutic potential of KME supplementation in type 2 diabetes is warranted.

**Trial registration:**

ClinicalTrials.gov NCT05518448.

**Funding:**

This project was supported by a Canadian Institutes of Health Research (CIHR) Project Grant (PJT-169116) and a Natural Sciences and Engineering Research Council (NSERC) Discovery Grant (RGPIN-2019-05204) awarded to JPL and an Exeter–UBCO Sports Health Science Fund Project Grant awarded to FBS and JPL.

**Graphical Abstract:**

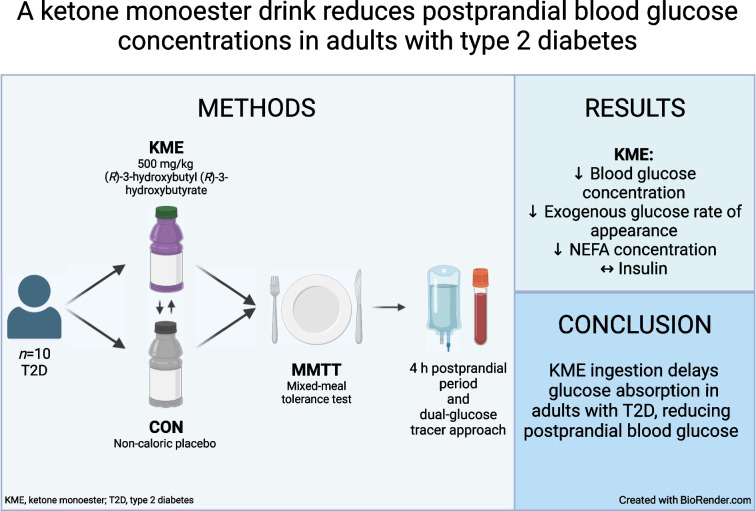

**Supplementary Information:**

The online version contains peer-reviewed but unedited supplementary material available at 10.1007/s00125-024-06122-7.


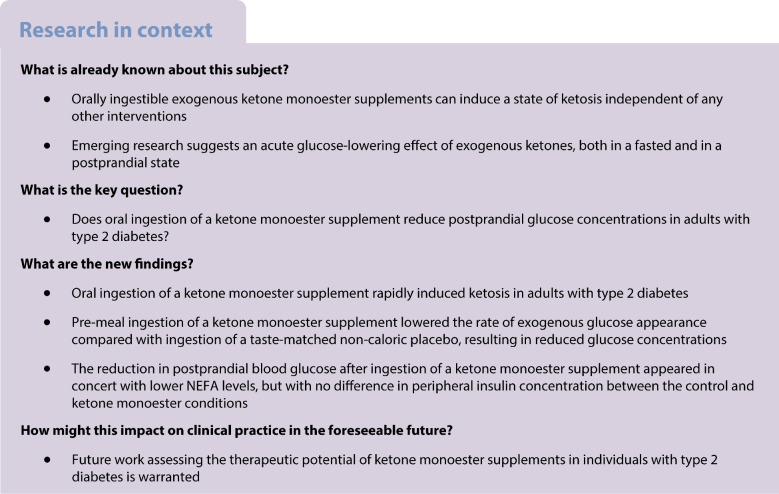


## Introduction

Oral administration of the ketone monoester (KME) (*R*)-3-hydroxybutyl (*R*)-3-hydroxybutyrate is a safe and tolerable method of inducing ketosis without the need to fast or restrict carbohydrates [1]. After ingestion, the KME is metabolised by gut and liver esterases into the active d isoform of β-hydroxybutyrate (β-OHB). A 20–50 g dose of KME typically increases circulating β-OHB from 0.1 mmol/l to ~2–4 mmol/l within ~30 min, which a recent systematic review and meta-analysis demonstrated to be consistently associated with an acute reduction in postprandial blood glucose (PBG) in individuals with and without impaired glucose tolerance [2].

Given that lowering PBG is a key treatment target in type 2 diabetes, the glucose-lowering capability of KME ingestion suggests therapeutic potential; however, this has never been tested directly. The aims of the present study were to determine whether KME ingestion can reduce PBG in individuals with type 2 diabetes and to explore the mechanisms involved. We applied dual-glucose stable isotope tracer techniques enabling assessment of the rate of glucose appearance from both oral/exogenous and endogenous sources, as well as the rate of glucose disappearance.

## Methods

### Participants

Ten adults diagnosed with type 2 diabetes (50% female, 59±1.7 years of age, HbA_1c_ 54±2 mmol/mol (7.1±0.2%); 6.4±1.6 years since diagnosis; *n*=4 taking metformin, *n*=1 gliclazide, *n*=1 pioglitazone) in the absence of other metabolic impairment or CVD participated in the present study. Detailed participant characteristics are presented in electronic supplementary material (ESM) Table 1. Exclusion criteria were lactose intolerance/milk allergies; blood pressure >160/100 mmHg; adhering to a ketogenic diet, undertaking periodic fasting or consuming ketone supplements; or smoking regularly. Ethical approval was obtained from the University of Exeter Sport and Health Sciences Ethics Committee in accordance with the Declaration of Helsinki and all participants provided written informed consent. The trial was registered on ClinicalTrials.gov (NCT05518448) and data collection was completed between April and November 2022 at the University of Exeter Nutritional Physiology Research Unit. We believe that our study sample is representative of the larger population of interest within the geographical location. Sex and ethnicity were self-reported; all participants identified as ‘white’ or ‘white British’.

### Experimental trials

The study used a double-blind, randomised, crossover design, with participants completing two experimental visits separated by ≥7 days (see ESM Methods, Randomisation). Participants were instructed to refrain from alcohol consumption and vigorous activity for 24 h before each visit (see ESM Methods, Pretesting). Participants arrived at the laboratory at 08:00±1 h following a 10 h overnight fast. A cannula was inserted into an antecubital vein to allow infusion of a [6,6-^2^H_2_]glucose tracer and two venous blood samples were taken 5 min apart to measure background isotopic enrichments. The infusion protocol began with a priming dose of [6,6-^2^H_2_]glucose (80×0.07 mg/kg body mass) administered as a bolus in 60 ml of 0.9% saline (wt/vol.; 154 mmol/l NaCl). Thereafter, from *t*=–120 min, the infusion was delivered at a continuous rate (0.07 mg kg body mass^−1^ min^−1^). A second cannula was inserted retrogradely into a dorsal hand vein and placed in a heated (55°C) unit to allow for arterialised venous blood sampling. Participants then consumed a KME or a taste-matched non-caloric placebo drink (CON) at *t*=–30 min. The KME drink contained 500 mg/kg body mass (*R*)-3-hydroxybutyl (*R*)-3-hydroxybutyrate (ΔG, TΔS, Thame, UK) mixed with 10 ml of calorie-free flavouring (Robinsons Mini, Britvic Soft Drinks, Dublin, Ireland) and water to a fluid volume of 100 ml. The placebo drink contained 250 µl of bitter agent (Mavala, Geneva, Switzerland) to flavour-match the KME, mixed with 10 ml of the same flavouring and water to a fluid volume of 100 ml. At *t*=0 min, participants were provided with a liquid mixed-meal tolerance test (MMTT) to be consumed within 5 min. The drink contained 1987.5 kJ of energy comprising 75 g of carbohydrate, 18 g of protein and 12 g of fat (see ESM Methods, Mixed-meal tolerance test). The MMTT was enriched with 1000 mg [U-^13^C_6_]glucose to allow for the calculation of the exogenous glucose appearance rate (RaE). The consumption of the MMTT marked the initiation of a 4 h postprandial period, during which arterialised venous blood samples were taken every 15 min for the first 2 h, followed by every 30 min for the final 2 h.

### Analysis

Sample size justification, blood sample collection and analyses and calculations used for the total glucose appearance rate (RaT), RaE, endogenous glucose production (EGP) and glucose disappearance rate (Rd) are presented in ESM Methods. Paired *t* tests were used to assess differences in summary measures (e.g. total glucose AUC and glucose variables in absolute terms). Two-way repeated measures ANOVAs (time × condition) were used to identify differences between conditions over time, with Sidak’s or Dunnett’s multiple comparisons tests performed where applicable. The pre-registered primary outcome measure was EGP. Secondary outcome measures were RaT and RaE, Rd, blood glucose, serum insulin, β-OHB and NEFA levels, and energy expenditure. Data are presented as means (±SEM) unless otherwise stated.

## Results

**Fig. 1:**
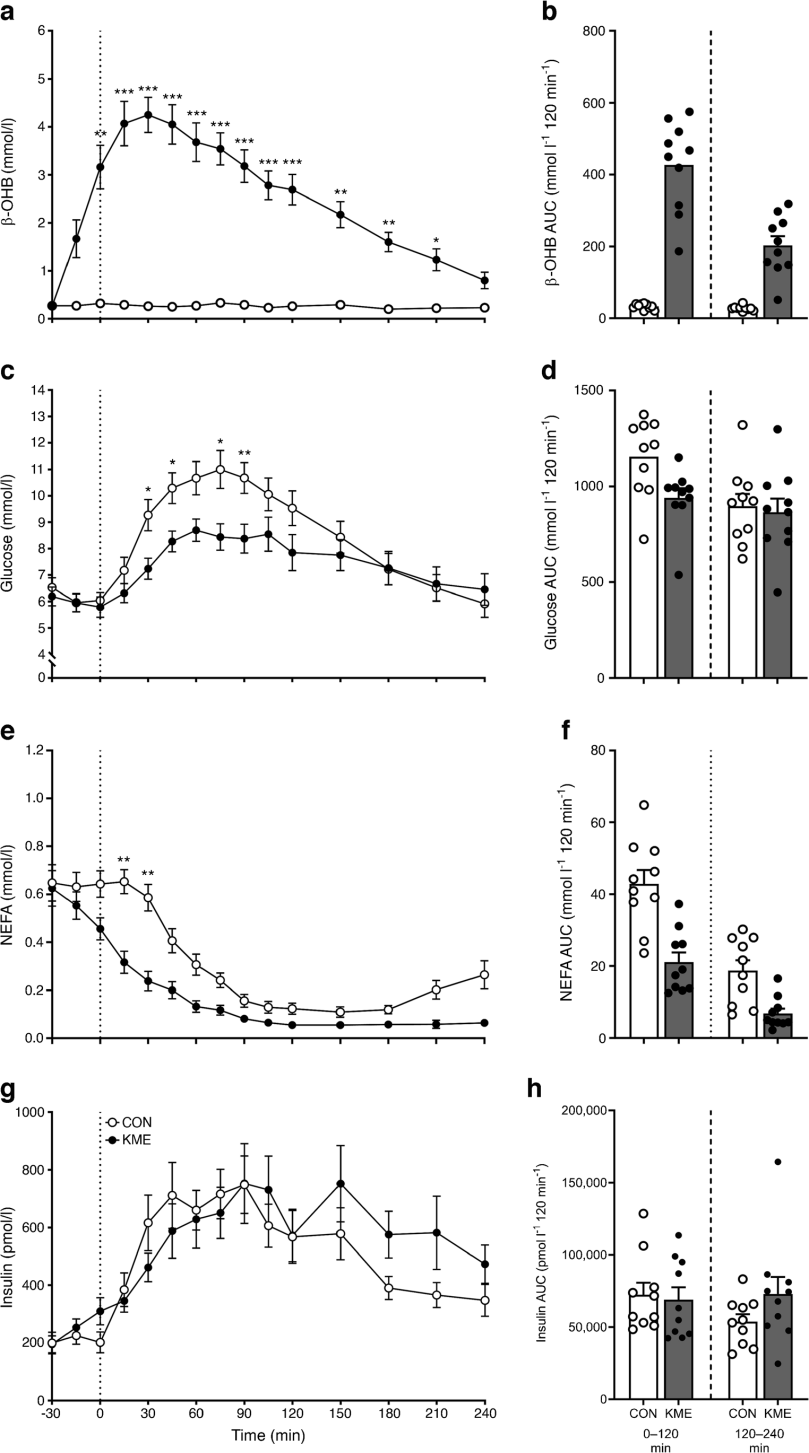
Time course (**a**, **c**, **e**, **g**) and AUC (**b**, **d**, **f**, **h**) of plasma β-OHB (**a**, **b**), blood glucose (**c**, **d**), serum NEFA (**e**, **f**) and serum insulin (**g**, **h**) concentrations in individuals with type 2 diabetes following the ingestion of the KME (*R*)-3-hydroxybutyl (*R*)-3-hydroxybutyrate (500 mg/kg body mass) or a CON preload and a subsequent MMTT (75 g carbohydrate, 18 g protein, 12 g fat). Data were analysed using two-way repeated measures ANOVA (time × condition), with Sidak’s or Dunnett’s multiple comparisons tests performed to detect differences at individual time points, and paired *t* tests performed to detect differences in AUC. (**b**, **d**, **f**, **h**) AUCs over 4 h are split into two epochs; 0–120 min (left) and 120–240 min (right), with individual participant values overlaid. Values are presented as means ± SEM. *p<0.05, **p<0.01, ***p<0.001 for difference between conditions at a given time point. (**a**) Time:* p*<0.0001; condition:* p*<0.0001; time × condition:* p*<0.0001. (**b**) Total:* p*<0.0001; 0–120 min:* p*<0.0001; 120–240 min:* p*=0.0001. (**c**) Time:* p*<0.0001; condition:* p*=0.0006; time × condition:* p*=0.0015. (**d**) Total:* p*=0.0017; 0–120 min:* p*=0.0003; 120–240 min:* p*=0.4651. (**e**) Time:* p*<0.0001; condition:* p*=0.0029; time × condition:* p*=0.0001. (**f**) Total:* p*=0.0011; 0–120 min:* p*=0.0007; 120–240 min:* p*=0.0051. (**g**) Time:* p*<0.0001; condition:* p*=0.2933; time × condition:* p*=0.0731. (**h**) Total:* p*=0.1366; 0–120 min:* p*=0.5449; 120–240 min:* p*=0.0353

Plasma β-OHB concentrations were significantly elevated 30 min after the ingestion of a KME supplement and remained elevated until 210 min, with a mean ± SEM of 3.0±1.0 mmol/l (Fig. 1a,b). Blood glucose concentrations following the MMTT increased to a lesser extent after KME ingestion, with a mean ± SEM of 8.7±0.5 mmol/l for CON and 7.5±0.3 mmol/l for KME (*p*<0.05). The postprandial glucose AUC was 12±3% lower for KME than CON, driven largely by an 18±3% lower AUC in the early postprandial period (0–120 min, both *p*<0.05; Fig. 1c,d). NEFA concentrations following the MMTT were suppressed to a greater extent after KME ingestion (*p*<0.01), resulting in a 49±11% reduction in the postprandial NEFA AUC for KME compared with CON (Fig. 1e,f). Insulin concentrations increased comparably under both conditions following the MMTT, with a mean ± SEM of 530±48 pmol/l for CON and 571±39 pmol/l for KME (*p*>0.05; Fig. 1g,h).

**Fig. 2:**
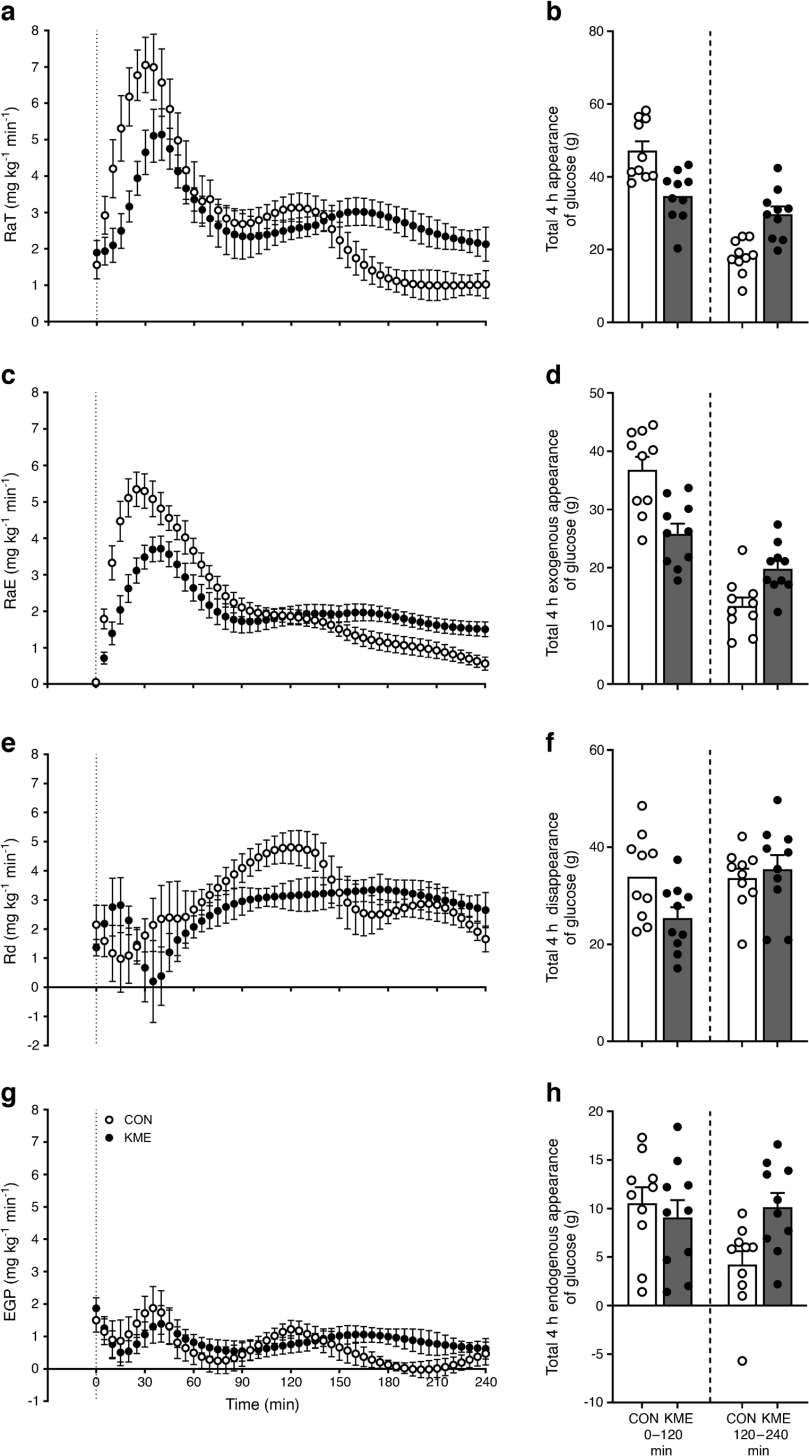
Time course of RaT (**a**, **b**), RaE (**c**, **d**), Rd (**e**, **f**) and EGP (**g**, **h**) in individuals with type 2 diabetes following the ingestion of the KME (*R*)-3-hydroxybutyl (*R*)-3-hydroxybutyrate (500 mg/kg body mass) or a CON preload and a subsequent MMTT (75 g carbohydrate, 18 g protein, 12 g fat). Glucose kinetics were calculated using the dual-glucose tracer method: continuous infusion of [6,6-^2^H_2_]glucose and the ingestion of oral [U-^13^C_6_]glucose in the MMTT. Data were analysed using two-way repeated measures ANOVA (time × condition) with Sidak’s or Dunnett’s multiple comparisons tests performed to detect differences at individual time points, and paired *t* tests performed to detect differences in absolute glucose appearance/disappearance. Appearance and disappearance over 4 h are split into two epochs; 0–120 min (left) and 120–240 min (right), with individual participant values overlaid. Values are presented as means ± SEM. (**a**) Time:* p*<0.0001; condition:* p*=0.0465; time × condition:* p*=0.0268. (**b**) Total: *p*=0.7452; 0–120 min:* p*=0.0028; 120–240 min:* p*=0.0060. (**c**) Time:* p*<0.0001; condition:* p*=0.0075; time × condition:* p*=0.0007. (**d**) Total: *p*=0.0750; 0–120 min:* p*=0.0015; 120–240 min:* p*=0.0266. (**e**) Time:* p*=0.0214; condition:* p*=0.0042; time × condition:* p*=0.2987. (**f**) Total: *p*=0.0207; 0–120 min:* p*=0.0096; 120–240 min:* p*=0.3520. (**g**) Time:* p*=0.0489; condition:* p*=0.3991; time × condition:* p*=0.5710. (**h**) Total:* p*=0.0843; 0–120 min:* p*=0.5724; 120–240 min:* p*=0.0180

Enrichment of [6,6-^2^H_2_]glucose decreased while enrichment of [U-^13^C_6_]glucose increased following the MMTT, with both occurring more markedly after ingestion of CON (ESM Fig. 1). RaT and, concomitantly, RaE increased to a lesser extent after ingestion of the KME than CON following the MMTT (Fig. 2a,c). RaT and RaE were lower after KME ingestion in the early postprandial period (0–120 min), which was reversed in the later postprandial period (120–240 min), resulting in no difference between conditions when calculated over the entire 4 h postprandial period (Fig. 2b,d). Rd following the MMTT increased to a greater extent after CON ingestion, which is reflected in a greater total glucose disappearance, largely driven by greater disappearance in the early postprandial period (when blood glucose concentrations were higher; Fig. 2e,f). There were no differences between conditions in either the rate of appearance of or total EGP following the MMTT (Fig. 2g,h). Specifically, mean ± SEM postprandial EGP was 0.76±0.15 and 0.88±0.10 mg kg^–1^ min^–1^ for CON and KME, respectively.

## Discussion

The present study demonstrates for the first time that KME ingestion prior to a meal can robustly reduce PBG in individuals with type 2 diabetes. There was a 28% reduction in the rate of glucose appearance over the first 2 h following meal ingestion, which resulted in a significant 18% reduction in PBG in the first 2 h and an overall 12% reduction in glucose AUC across the entire 4 h. Contrary to our hypothesis, EGP was unaffected by KME ingestion and played no significant role in reducing postprandial glucose concentrations. The postprandial glucose-lowering effect seen here was greater than that observed in individuals with normal [3, 4] or impaired glucose tolerance [5], indicating that the mechanism(s) of action for pre-meal KME-mediated glucose reduction appear to be intact in individuals with type 2 diabetes.

The mechanism(s) by which KME reduced PBG fits with a delay in, rather than complete inhibition of, glucose absorption, as total glucose appearance across the entire 4 h postprandial period was unaltered due to a greater RaT in the later postprandial period compared with the control condition. The delay could be due to slowed gastric emptying; however, the effect of KME ingestion on gastric emptying remains inconclusive [6, 7]. Similarly, it has been repeatedly shown that meal preloads increase the secretion of gut peptides, influencing pancreatic hormone secretion and modulating gastrointestinal function [6, 7]. As such, further research on the effects of KME ingestion on gastrointestinal transit and function (including hormonal secretion and gastric emptying) is warranted. In this regard, it should be noted that the placebo in our study contained no caloric value; future work should compare KME with other energy-matched meal preloads. Additionally, bitter taste-sensing type 2 receptors (TAS2Rs) are thought to play a role in regulating PBG via upregulated glucagon-like peptide-1 signalling [8] and it is possible that the bitterant in the control condition may have decreased PBG and dampened the differences between conditions*.* Furthermore, the potential for metformin or other glucose-lowering medication to interact with the effect of KME on reducing, or delaying, PBG warrants further investigation.

We also report a potent reduction in circulating NEFA concentrations after KME ingestion compared with CON ingestion in individuals with type 2 diabetes, in the absence of a difference in circulating insulin levels between conditions. While insulin concentrations did not differ between conditions, this is not necessarily indicative of identical insulin secretion or clearance in both conditions. The observed reduction in NEFA levels fits with a role of β-OHB as an endogenous ligand for the hydroxycarboxylic acid receptor 2 (HCAR2) [9], which inhibits adipose tissue lipolysis; however, we did not employ a tracer to directly measure the rate of lipolysis, which has been performed previously in healthy individuals [10]. Pharmacologically manipulating circulating NEFA levels in humans can rapidly modulate insulin-stimulated glucose disposal in skeletal muscle [10] and EGP in the liver [11], so it is surprising that KME ingestion reduced Rd but did not measurably reduce EGP. This is particularly notable given previous observations of reduced EGP when circulating β-OHB was increased via oral ketone administration in healthy individuals [12, 13]. The lack of suppression of EGP with KME in our study could be due to measurement error, which could be reduced by using more advanced computational methods with variable tracer infusions to mimic the expected effect. However, approaches similar to ours have previously yielded accurate results [14] and detected differences in EGP with pharmacological intervention in individuals with type 2 diabetes [15].

We acknowledge several other caveats when interpreting our results. First, the postprandial period we investigated was in the context of participants breaking their fast and it is unclear whether the PBG-lowering effect would be retained for subsequent meals and how it might be modulated by circadian fluctuations in metabolism. Additionally, the observed lowering of PBG, although marked, was relatively short-lived, occurring only in the initial postprandial period. Finally, although we included both sexes in our study, we were not powered in this proof-of-concept trial to determine if (and to what extent) biological sex has the potential to moderate the observed effect, which should be considered in future investigations.

In conclusion, the ingestion of a KME preload prior to a MMTT led to a robust decrease in PBG in individuals with type 2 diabetes. Clinical trials to assess the efficacy of daily KME ingestion as a treatment option for type 2 diabetes are warranted.

### Supplementary Information

Below is the link to the electronic supplementary material.

Supplementary file1 (PDF 372 KB)

## Data Availability

Data that support the findings of this study are available from the corresponding author on reasonable request.

## Abbreviations

CON: Non-caloric placebo drink
EGP: Endogenous glucose production
KME: Ketone monoester
MMTT: Mixed-meal tolerance test
β-OHB: β-hydroxybutyrate
PBG: Postprandial blood glucose
RaE: Exogenous glucose appearance rate
RaT: Total glucose appearance rate
Rd: Glucose disappearance rate
