# Supplementary material for: A ketone monoester drink reduces postprandial blood glucose concentrations in adults with type 2 diabetes: a randomised controlled trial
Source: Diabetologia. 2024 Mar 14;67(6):1107–13. doi: 10.1007/s00125-024-06122-7 (PMC11058041; doi:10.1007/s00125-024-06122-7)
Supplement: Supplementary file 1 — Supplementary file1 (PDF 372 KB) [file 125_2024_6122_MOESM1_ESM.pdf]

Supplementary Material

Index

	**Page**
Methodology	2
Randomisation	2
Pre-testing	2
Mixed-Meal Tolerance Test	3
Sample size	3
Blood sample collection and analysis	3
Calculations	5
References	7
Supplementary Table 1	8
Supplementary Figure 1	9

Methodology

Randomisation

To ensure blinding, the lead investigator generated a randomisation sequence using the Excel 'RAND' function, which was then handed to a member of research staff who was not associated with experimental testing. This researcher then assigned the sequence to conditions, keeping the resultant document under password protection. Preload drinks were then prepared by another member of research staff not associated with experimental testing. The blinding sequence was only revealed once data collection analysis for the study was complete.

Pretesting

Prior to inclusion in the study, participants completed a screening visit during which body mass, height, and blood pressure were recorded, and a fasting blood sample was collected to assess circulating glycated haemoglobin and fasting plasma glucose. Participants were given a 24-hour food log to complete on the day before their first experimental trial, with the instruction that this would then need to be replicated before their second experimental trial. Participants were instructed to avoid foods with naturally high enrichments of the Carbon-13 isotope (^{13}C) in the three days before each trial, namely: maize, pineapple, sugar cane, millet, purslane, tropical fruits, canned foods, tequila, and some fish (trout, haddock, tuna, whiting). Participants were also provided with a three-day diet diary to record their habitual dietary intake for two weekdays and one weekend day. Participants were instructed to abstain from taking their medication at least 12 h prior to each experimental trial.

Mixed-Meal Tolerance Test

The mixed-meal tolerance test (MMTT) contained 250 mL whole milk, 50 g dextrose, 18.7 g skimmed milk powder, and 9 g of full fat milk powder.

Sample size

A priori sample size estimate was based on previous work that demonstrated a reduction in the incremental area under the postprandial blood glucose curve with ketone monoester supplementation compared to placebo in response to glucose ingestion in adults with obesity (1). Based on these data, we assumed an effect size of d=1.0, which we considered a large and clinically relevant reduction in blood glucose for this preliminary study. The a priori primary outcome was defined as the rate of endogenous glucose production (EGP), which can only be captured using glucose tracers, is a strong predictor of the postprandial glucose response in people living with type 2 diabetes (2), and we hypothesized played a key role in the glucose-lowering mechanisms of elevated β-OHB (3). A sample size calculation (G*Power v3.1) for a paired t-test estimated a sample size of 10 was required for 80% power to detect a two-tailed difference at a significance level of 0.05 with an effect size of d=1.0.

Blood Sample Collection and Analyses

Ten mL of arterialised-venous blood (with the exception of the venous samples at baseline) were collected into a syringe at each time point. A 20 µL plastic capillary tube was immediately filled with whole blood and analysed for blood glucose concentrations (Biosen C-Line GP+). Five mL of that sample were added to EDTA-containing tubes (BD vacutainer LH; BD Diagnostics, Nu-Care) and centrifuged for 10 min at 4000 rpm at 4 °C. The plasma supernatant was then removed, aliquoted, and stored at -80 °C for subsequent analyses. The remaining 5 mL of blood were added to serum-separating Silica containing vacutainers (BD

vacutainers SST II, BD Diagnostics, Nu-Care) and left upright to clot at room temperature for 30 min before being centrifuged for 10 min at 4000 rpm at 4 °C. The serum supernatant was then removed, aliquoted, and stored at -80°C for subsequent analyses.

Serum insulin and non-esterified fatty acid (NEFA) concentrations were analysed using commercially available assay kits (DRG Insulin ELISA, EIA-2935, DRG International Inc., and Randox NEFA Assay, FA 115, Randox Laboraties Ltd.). Plasma β-OHB was measured using a commercially available kit (Cayman Chemicals, Ann Arbor, MI). All the samples were batch analysed after data collection was completed, and all the samples for a given participant were run within the same assay.

Enrichments of intravenously and orally administered [6,6-$^{2}H_{2}$]glucose and [U-$^{13}C_{6}$]glucose, respectively, were determined in the collected plasma samples by gas chromatography-mass spectrometry (GC-MS). Samples were deproteinised by adding 300 μL of ice-cold methanol to 50 μL of plasma and placed on ice for 30 min. Following centrifugation for 12 min at 12000 g at 4 ºC, 300 μL of supernatant were transferred to a glass screw-neck vial and air-dried. Anhydride:pyridine (2:1) was added to the air-dried samples, vortexed, and left at room temperature for 30 min to form a penta-acetate glucose derivative. The samples were then analysed by GC-MS (6890N GC coupled with a 5975B MSD; Agilent Technlogies) in duplicate using electron impact ionisation and selected ion monitoring for measurement of isotope ratios. One μL of the sample was injected in splitless mode (injector temperature of 280 °C). Peaks were resolved using an HP5-MS 30 m × 0.25 mm ID × 0.25 μm capillary column (Agilent). Helium was used as carrier gas at 1.2 mL·min^{-1} constant flow rate. The temperature ramp was set from 80 – 260 °C at 15 °C·min^{-1}. Selected ion recording conditions were used to monitor fragments m/z 200, 202 and 205 for glucose.

Calculations

Total rate of glucose appearance (RaT) ($mg \cdot kg^{-1} \cdot min^{-1}$) (Eq. 1) was calculated by using the non-steady state equation of Steele as adapted by De Bodo (4), with a value of 0.23 $L \cdot kg^{-1}$ for volume of distribution (V) as proposed by Livesey, and a pool fraction value of 1 (5). $F_{[6,6-^2H_2]-glucose}$: is the [6,6-$^2H_2$]-glucose infusion rate (0.07 $mg \cdot kg^{-1} \cdot min^{-1}$), p is the pool fraction (1L), 180.16 is the molecular weight of glucose ($g \cdot mol^{-1}$), C_1 and C_2 are the glucose concentrations at t_1 and t_2 ($mmol \cdot L^{-1}$), respectively, $IE1_{[6,6-^2H_2]-glucose}$ and $IE2_{[6,6-^2H_2]-glucose}$ are the plasma [6,6-$^2H_2$]-glucose isotopic enrichments at t_1 and t_2, respectively (mol % excess (MPE)), and t_1 and t_2 are the first and second collection time points (min), respectively.

Eq.1: $RaT =$

$$\frac{F_{[6,6-^2H_2]-glucose} - pV \cdot 180.16 \cdot \frac{(C_1 + C_2)}{2} \cdot \left(\frac{IE2_{[6,6-^2H_2]-glucose} - IE1_{[6,6-^2H_2]-glucose}}{t_2 - t_1}\right)}{\left(\frac{IE1_{[6,6-^2H_2]-glucose} + IE2_{[6,6-^2H_2]-glucose}}{2}\right)}$$

Exogenous rate of glucose appearance (RaE) ($mg \cdot kg^{-1} \cdot min^{-1}$) (Eq. 2) was calculated using Steele's equation for non-steady state (6) as adapted by Schneiter, where $\boldsymbol{F_{infusion}}$ is the total glucose infusion rate (0.07 $mg \cdot kg^{-1} \cdot min^{-1}$), V is the volume of distribution for glucose (0.23 $L \cdot kg^{-1} \cdot min^{-1}$), $IE1_{[13C_6]-glucose}$ and $IE2_{[13C_6]-glucose}$ are the plasma [$13C_6$]-glucose isotopic enrichment at t_1 and t_2 (mol % excess (MPE)), respectively, [U-$^{13}C_6$]glucose is the amount of [U-$^{13}C_6$]glucose ingested (g) and glucose is the total amount (labelled and unlabelled) of glucose ingested (g).

Eq. 2: $RaE =$

$$\frac{(RaT - F_{infusion}) \cdot \left(\frac{IE1_{[13C_6]-glucose} + IE2_{[U-13C_6]-glucose}}{2}\right) + pV \cdot 180.16 \cdot \frac{(C_1 + C_2)}{2} \cdot \left(\frac{IE2_{[U-13C_6]-glucose} - IE1_{[U-13C_6]-glucose}}{t_2 - t_1}\right)}{\left(\frac{[U-13C_6]-glucose}{glucose}\right)}$$

Endogenous glucose production (EGP) was calculated as RaT - RaE. Identical behaviour of labelled and unlabelled glucose molecules was assumed throughout, and the baseline sample for each trial was used to account for background isotopic enrichments. After calculating RaT, Rd,and RaE for each time-point, data were then smoothed using Graphpad Prism 9. A spline curve was fitted to each individual's data between the time periods; 0-60 min, 60-180 min and 180-240 min (7). The program interpolated at time intervals of 1 min which was used to calculate individual total appearance and disappearance of glucose in grams (0-120 min, 120-240 min, and 0-240 min). EGP was calculated using the interpolated data from RaT and RaE. These data were then fitted with a spline curve as described.

References

1. Myette-Côté É, Caldwell HG, Ainslie PN, Clarke K, Little JP. A ketone monoester drink reduces the glycemic response to an oral glucose challenge in individuals with obesity: a randomized trial. The American Journal of Clinical Nutrition 2019;110:1491-1501
2. Rizza RA. Pathogenesis of Fasting and Postprandial Hyperglycemia in Type 2 Diabetes: Implications for Therapy. Diabetes 2010;59:2697-2707
3. Falkenhain K, Islam H, Little JP. Exogenous ketone supplementation: an emerging tool for physiologists with potential as a metabolic therapy. Experimental Physiology 2023;108:177-187
4. Debodo RC, Steele R, Altszuler N, Dunn A, Bishop JS. ON THE HORMONAL REGULATION OF CARBOHYDRATE METABOLISM; STUDIES WITH C14 GLUCOSE. Recent Prog Horm Res 1963;19:445-488
5. Livesey G, Wilson PDG, Dainty JR, Brown JC, Faulks RM, Roe MA, Newman TA, Eagles J, Mellon FA, Greenwood RH. Simultaneous time-varying systemic appearance of oral and hepatic glucose in adults monitored with stable isotopes. American Journal of Physiology-Endocrinology and Metabolism 1998;275:E717-E728
6. Steele R, Wall JS, Bodo RCd, Altszuler N. Measurement of Size and Turnover Rate of Body Glucose Pool by the Isotope Dilution Method. American Journal of Physiology-Legacy Content 1956;187:15-24
7. Wolfe RR, Chinkes DL. *Isotope tracers in metabolic research: principles and practice of kinetic analysis*. John Wiley & Sons, 2005

ESM Table 1. Participant Characteristics

Participant Characteristics, mean ± SEM	
Age (y)	59 ± 1.7
Sex (m/f)	5/5
Body mass (kg)	95 ± 4.5
BMI ($kg \cdot m^{-2}$)	32.0 ± 1.0
Systolic Blood pressure (mmHg)	129 ± 3.7
Diastolic Blood pressure (mmHg)	75 ± 3.4
HbA_{1c} (mmol/l)	54 ± 2.4
Fasting plasma glucose (mmol/l)	7.6 ± 0.5
Diagnosed (y)	6.4 ± 1.6
**Diabetes Medication, N (%)**	
Metformin	4 (40)
Gliclazide	1 (10)
Pioglitazone	1 (10)
**Habitual Dietary Intake, mean ± SEM**	
Energy ($MJ \cdot d^{-1}$)	8.7 ± 0.9
Energy ($kcal \cdot d^{-1}$)	2091 ± 230.7
Protein ($g \cdot d^{-1}$)	95 ± 9.0
Protein ($g \cdot kg^{-1}$ $BM \cdot d^{-1}$)	1.0 ± 0.1
Carbohydrate ($g \cdot d^{-1}$)	186 ± 25.4
Fat ($g \cdot d^{-1}$)	97 ± 12.5
Fibre ($g \cdot d^{-1}$)	28 ± 5.8

ESM Figure 1

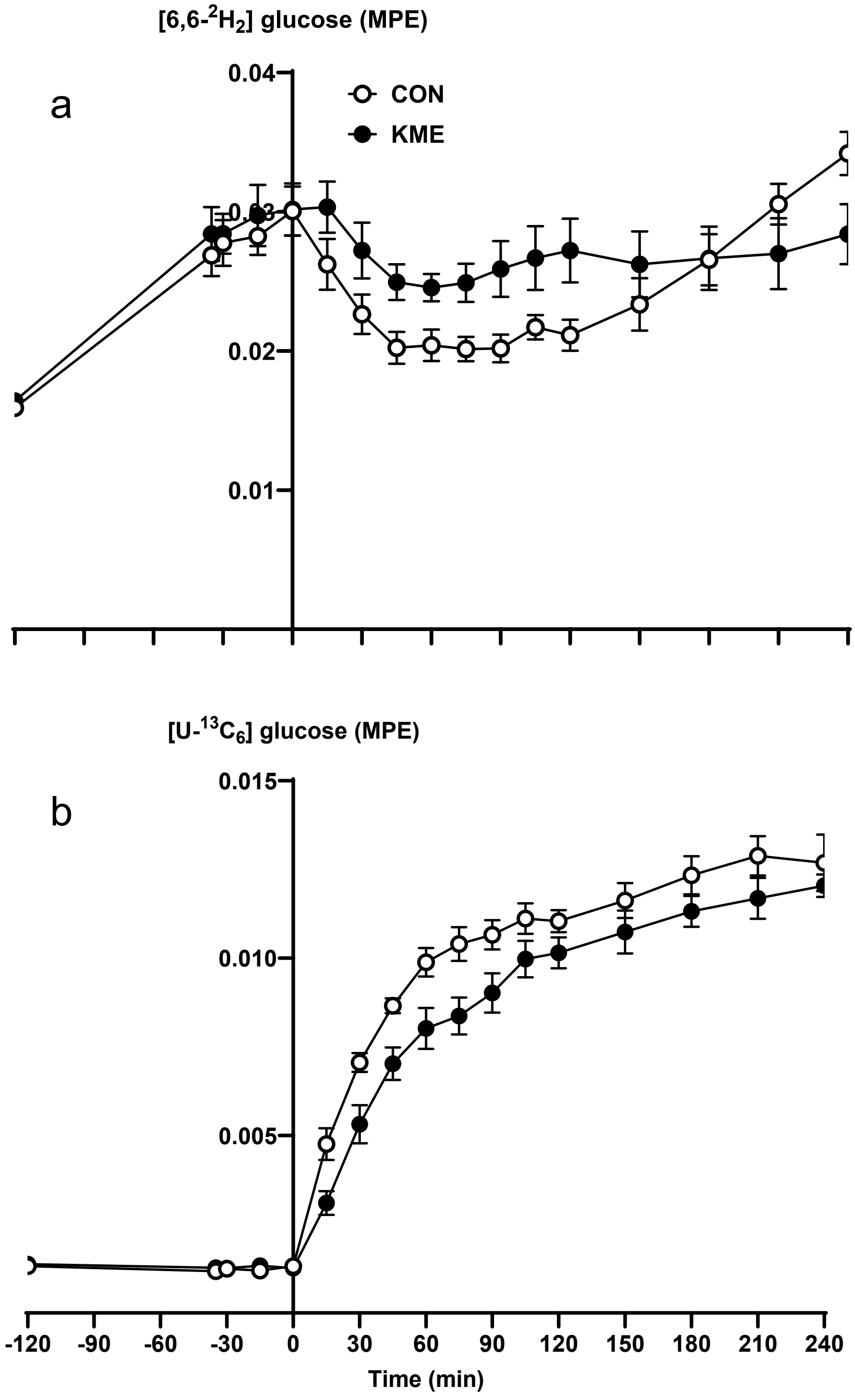

Time course of [U-$^{13}C_6$]glucose (A) and [6,6-$^2H_2$]glucose (B) enrichments following the ingestion of oral [U-$^{13}C_6$]glucose in a mixed meal tolerance test (MMTT; 75 g carbohydrate, 18 g protein, 12 g fat) and the primed-continuous infusion of [6,6-$^2H_2$]glucose. Data were analysed using two-way repeated measures ANOVA (time × condition). Values are mean ±

SEM. MPE, mol % excess. a; time: $p < 0.0001$, condition: $p = 0.0163$, time x condition: $p = 0.0004$. a; time: $p < 0.0001$, condition: $p = 0.0021$, time x condition: $p = 0.0358$.
